# Oxygen Venous Embolism After Hydrogen Peroxide Use During Percutaneous Nephrolithotomy

**DOI:** 10.1089/cren.2018.0111

**Published:** 2019-03-18

**Authors:** John Michael DiBianco, Jessica Lange, Daniel Heidenberg, Patrick Mufarrij

**Affiliations:** ^1^ Department of Urology, George Washington University Medical School, Washington, District of Columbia.

**Keywords:** nephrolithiasis, percutaneous nephrolithotomy, oxygen pulmonary embolus, hydrogen peroxide

## Abstract

***Background:*** Hydrogen peroxide (H_2_O_2_) is a common antiseptic that is available without a prescription in the United States, and it is indicated for minor dermal abrasion; mouth, gum, or dental irritations; and removal of oral secretion. Several other medical uses have also been described, including clot dissolution during endoscopic gastrointestinal evaluation, cleansing of orthopedic surgical sites, and bladder irrigation. However, these uses of H_2_O_2_, as well as high-dose ingestion, have been associated with a wide variety of medical complications, including but not limited to air pulmonary embolism and stroke.

***Case Presentation:*** Our patient is a 51-year-old female with a medical history of hypertension, familial, hypercholesterolemia, gallstones, depression, coronary artery disease (identified on calcium study because of familial hypercholesterolemia), nephrolithiasis, and recurrent cystitis. She required percutaneous nephrolithotomy and had H_2_O_2_ administered for clot dissolution. The clinical and temporal evidence would suggest a transient pulmonary air embolus after the intrarenal administration of or irrigation with H_2_O_2_, large amounts under high pressure.

***Conclusion:*** This represents the first reported incidence of air embolus as a result of intrarenal administration of H_2_O_2_.

## Introduction

Hydrogen peroxide (H_2_O_2_) is a common antiseptic that is available without a prescription in the United States, and it is indicated for minor dermal abrasion; mouth, gum, or dental irritations; and removal of oral secretion.^1^ Its mechanism of action involves the release of oxygen and water upon tissue contact. The complications of these uses are minor and mostly include pain on administration and irritation to areas of administration.^1^

Several other medical uses have also been described, including clot dissolution during endoscopic gastrointestinal evaluation, cleansing of orthopedic surgical sites, and bladder irrigation. However, these uses of H_2_O_2_, as well as high-dose ingestion, have been associated with a wide variety of medical complications, including but not limited to air pulmonary embolism and stroke.^2–5^

## Case Report

**FIG. 1.**
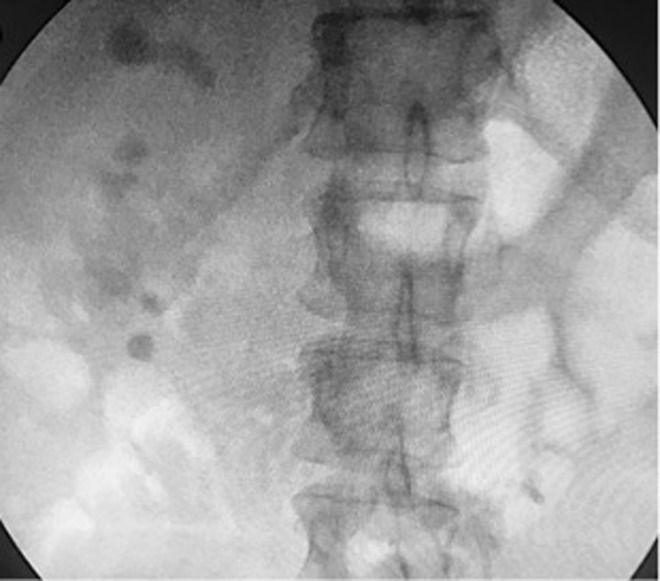
Preoperative stone burden.

Our patient is a 51-year-old female with a medical history of hypertention, familial, hypercholesterolemia, gallstones, depression, coronary artery disease (identified on calcium study because of familial hypercholesterolemia), nephrolithiasis, and recurrent cystitis. Her first stone event was in 2008 that subsequently required ureteroscopy (URS) and ureteral stent placement. She presented to our outpatient clinic initially in consultation for left-sided abdominal pain, and subsequent CT scan revealed nephrolithiasis with three separate left ureteral stones (Fig. 1), requiring urgent ureteral stent placement.

After urgent decompression with a stent, she was scheduled for bilateral URS with laser lithotripsy (LL) and possible bilateral percutaneous nephrolithotomy (PCNL) for definitive stone management. She ultimately underwent bilateral URS with LL and right-sided PCNL. Postoperative CT scan revealed residual right-sided stone burden, so she was scheduled for a second stage right-sided PCNL. In the interim, her postoperative course was complicated by gross hematuria and anemia, requiring transfusion of 2 U of packed red blood cells.

**FIG. 2.**
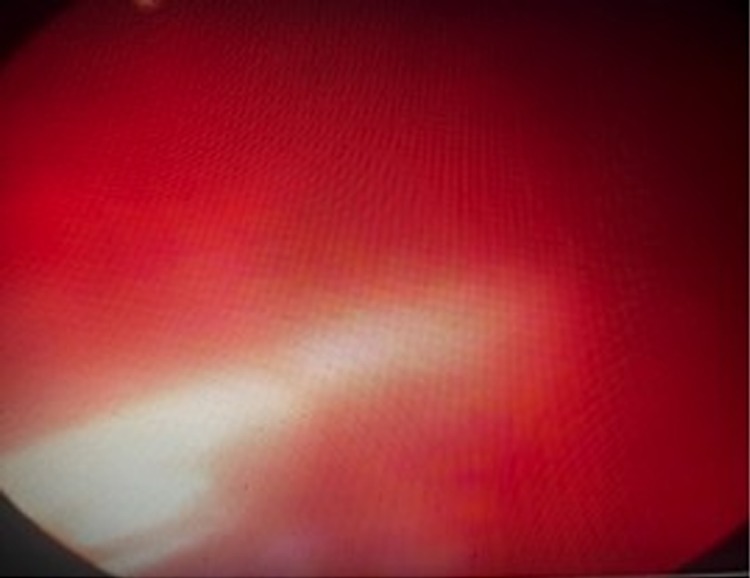
Poor intraoperative observation.

**FIG. 3.**
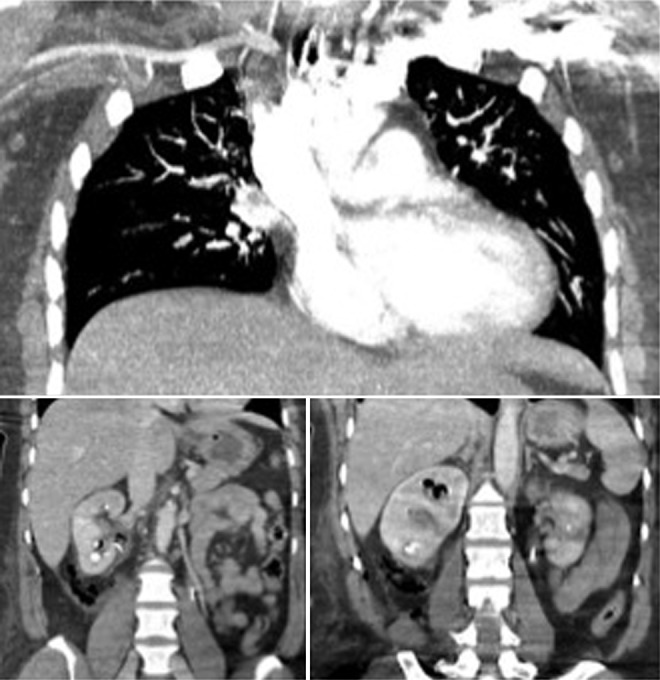
Intraoperative CT angiography.

During her planned second stage PCNL, initial ureteroscopic observation was very poor because of extensive clot burden in the right renal pelvis and calices (Fig. 2). Three percent H_2_O_2_ standard preparation was diluted three times and ∼100 mL was instilled using a 60 mL Toomey syringe through the current indwelling 22 Councill nephrostomy tube to dissolve the extensive clot burden and improve observation. Approximately 45 seconds after instillation, the anesthesiologist noted that the patient's end tidal carbon dioxide (CO_2_) had suddenly plummeted (0–9) and she had become hypotensive. ST segment elevation was also noted on the telemetry tracings that were a change from her preoperative electrocardiogram. Owing to immediate concern for pulmonary embolus, the procedure was terminated, and the patient was taken out of dorsal lithotomy position and placed in the supine position. The patient's blood pressure increased over the next several minutes, and she became hemodynamically stable; however, her end tidal CO_2_ remained well below normal. She was immediately transferred from the operating room to radiology for emergent CT angiography (CTA). At the time of CTA, the patient's end tidal CO_2_ had risen to 30 to 40. Ultimately the CTA revealed no evidence of pulmonary thrombus; however, abdominal follow-through was notable for extensive air in the right renal collecting system (Fig. 3). The patient was transferred back to the operating room, extubated, and brought to postanesthesia care unit for intensivist consultation without complication. She demonstrated stability and returned to her inpatient floor. She recovered well without evidence of residual effects. Three days later, she underwent her final stage procedure to render her stone free without complications. Final postoperative CT after third stage procedure revealed no residual stones bilaterally.

## Discussion

Intraoperative pulmonary embolus is hallmarked by rapid respiratory distress, fall in end tidal CO_2_, and possible hemodynamic instability, depending on the size of the embolus. It is estimated that detrimental effects of venous gas embolism are determined by the total volume of air injected as well as the rate of injection and the final location of the air embolus in the pulmonary arterial tree. It is estimated that 300 to 500 mL of gas introduced at a rate of 100 mL/sec can be acutely fatal for humans.^6^

The clinical and temporal evidence in this case would suggest a transient pulmonary air embolus after the intrarenal administration of H_2_O_2_. It is not known whether H_2_O_2_ administration through an indwelling nephrostomy tube during URS can produce these levels of intravascular gas. A thorough search of the available literature did not reveal any prior reports of this phenomenon; therefore, we believe we have presented the first case here. Given the patient's persistent hematuria and anemia, it is likely that she had increased intravascular H_2_O_2_.

Studies suggest that excessive H_2_O_2_ under pressure may result in peroxynitrite production that vascular endothelium cannot metabolize rapidly, resulting in its disruption and subsequent air embolism.^2^ Case reports demonstrate that intraoperative usage of H_2_O_2_ for spinal, gastroenterology, and neurosurgical surgeries have resulted in venous air embolism with metabolic disturbances. These typically involve the use of copious amounts of H_2_O_2_.^4^ There is even a risk of air embolism with H_2_O_2_ injestion.^3^

In general, if an intraoperative air embolus is suspected, assessment for air stability and vascular access is of the utmost priority. At the same time, the patient should be positioned in the left lateral decubitus position (Durant's maneuver) with or without head down to avoid further embolization. Next, attention should be turned to determining hemodynamic stability.^6,7^ Hemodynamically stable patients without end organ damage can be treated with high-flow oxygen and repositioning, whereas hemodynamically unstable patients require more aggressive treatment.^7^ Central venous access should be obtained in those in whom peripheral access cannot be obtained, and in those who need infusions of large volumes of fluids, infusion of vasopressors, and/or frequent blood draws. Additional treatments include hyperbaric oxygen, withdrawal of air from the right atrium, and cardiac massage in extreme cases.^6,7^

We believe that the intraoperative instability and clinical decompensation along with drop in end tidal CO_2_ in our case likely resulted from transient intraoperative usage of H_2_O_2_ with moderate irrigating force in a closed cavity. In addition, given the patient's preoperative evidence of hemorrhage, she may have had an increased risk of venous backflow. We consider our experience in addition to the literature to date as adequate evidence for advising caution when considering its use during urologic procedures.

## Conclusion

The clinical and temporal evidence would suggest a transient pulmonary air embolus after the intrarenal administration of or irrigation with H_2_O_2_, large amounts under high pressure. This represents the first reported incidence of air embolus as a result of intrarenal administration of H_2_O_2_.

## Abbreviations Used

CT: computed tomography
CTA: CT angiography
CO_2_: carbon dioxide
H_2_O_2_: hydrogen peroxide
LL: laser lithotripsy
PCNL: percutaneous nephrolithotomy
URS: ureteroscopy
